# Female Genital Mutilation: Knowledge and Skills of Health Professionals

**DOI:** 10.3390/healthcare9080974

**Published:** 2021-07-31

**Authors:** Brígida Molina-Gallego, Laura Mordillo-Mateos, Gonzalo Melgar de Corral, Sagrario Gómez-Cantarino, Begoña Polonio-López, M Idoia Ugarte-Gurrutxaga

**Affiliations:** 1 Faculty of Physiotherapy and Nursing, Campus Toledo, University of Castilla-La Mancha, 45004 Toledo, Spain; Brigida.Molina@uclm.es (B.M.-G.); Gonzalo.Melgar@uclm.es (G.M.d.C.); Sagrario.Gomez@uclm.es (S.G.-C.); Maria.Ugarte@uclm.es (M.I.U.-G.); 2 Faculty of Health Sciences, Universidad de Castilla la Mancha, 45600 Talavera de la Reina (Toledo), Spain; Begona.Polonio@uclm.es; 3 Health Sciences Research Unit: Nursing (UICISA: E), Coimbra School of Nursing (ESEnfC), 3004-011 Coimbra, Portugal

**Keywords:** female genital mutilation, healthcare professionals, women’s health

## Abstract

Background: Female genital mutilation (FGM) is any process that injures female genitals for non-medical reasons and is a violation of women’s human rights. An important number of women from countries where FGM is performed are arriving to Western countries. Health professionals are important for detecting cases of FGM. No surveys to assess knowledge, attitudes and practices on FGM among healthcare professionals has been conducted in Castilla la Mancha (Spain) until now. Methods: The main goal of the study is assessing knowledge, attitudes and perceptions of healthcare professionals in relation to FGM. A cross-sectional descriptive study was conducted based on self-administered online surveys to nurses, midwives, family doctors, pediatricians, obstetrics and gynecologists. Results: In total, 1168 professionals answered the surveys. Just 13.9% indicated that they had received training in FGM, however just 10.7% correctly identified the three types of FGM, 10.7% the countries where it is usually practiced, 33.9% knew the legislation in Spain and only 4.4% found a case of FGM during their professional practice. Regarding the knowledge about protocols, 8.64% of the sample indicated to know one of them. Conclusion: The present study demonstrate that it is necessary to improve the training and awareness of healthcare professionals related to FGM in Castilla la Mancha.

## 1. Introduction

Female genital mutilation (FGM) is any process that injures the female genital organs or the partial or complete removal of the external genitalia of a woman or girl for non-medical reasons, and is a violation of women’s human rights. [1,2]. Up to 200 million girls worldwide have endured FGM [3].

A study of more than 28,000 women in six African countries found FGM caused adverse obstetric outcomes including disease and death [4,5], emphasizing that FGM is also a growing health issue across Europe, although the practice is illegal in most European countries [6,7]. 

The Royal College of Nursing (RCN) (2016) advises that nurses need to be aware of best practice when dealing with FGM; this includes knowing what FGM is, where it is carried out, how to prevent it and how women who have undergone it can be supported physically and emotionally [3]. Andro and colleagues found that health professionals need better education on FGM [5]. Relph and collaborators [8] found that fewer than 25% of practitioners had formal training on FGM, even in areas of high prevalence of FGM. Additionally, a large study developed by Zaidi et al. (2007) found that health professionals had substantial gaps in their knowledge regarding how to deal with FGM, and emphasized the need for appropriate FGM training [8,9].

Female genital mutilation comprises all procedures that involve partial or total removal of the external female genitalia or injury to the female genital organs for non-medical reasons. Healthcare providers for women and girls living with female genital mutilation have reported difficulties in recognizing, classifying, and recording female genital mutilation, which can adversely affect treatment of complications and discussions of the prevention of the practice in future generations. According to the World Health Organization, female genital mutilation is classified into four types, subdivided into subtypes (Table 1). An agreed-upon classification of female genital mutilation is important for clinical practice, management, recording, and reporting, as well as for research on prevalence, trends, and consequences of female genital mutilation. It provides a visual reference and learning tool for healthcare professionals that can be consulted by caregivers when unsure on the type of female genital mutilation diagnosed and used for training and surveys for monitoring the prevalence of female genital mutilation types and subtypes [10,11].

Regarding epidemiology, the WHO estimates that some 140 million women and girls suffer the consequences of FGM. It is usually performed on girls between 4 and 14 years of age, although it is also performed shortly after birth, on women who are going to marry, during their first pregnancy or after childbirth [12].

The consequences suffered by women and girls who undergo FGM can be physical, psychological and social. Among the physical consequences we distinguish the immediate ones, which are those that occur when the mutilation is being performed, and those of the medium-long term, those that appear after performing the technique. In the short term, severe pain, bleeding, infections, lesions in the organs and the affected area and fractures may appear, and in the medium-long term: anemia, complications in childbirth, depression, fear and even death. The social consequences are related to the rejection that the girl or the woman may suffer [12,13,14,15,16,17].

FGM is practiced mainly in 29 countries in sub-Saharan Africa and the Middle East. In Africa, there are 92 million women and girls over the age of 10 who have already been mutilated. If this continues, it is estimated that in the next decade 30 million girls may be at risk of mutilation. Due to the process of globalization and the constant flow of migration, we find that FGM has spread to Australia, New Zealand, Europe, the United States and Canada [18,19].

Due to this geographic dispersion of FGM, the international community has taken action in this regard. FGM is considered a violation of human and legal rights of women and constitutes an ethical dilemma for healthcare professionals.

Specifically, in Spain, the legislation in this regard considers FGM as a crime, included in the Criminal Code as a crime of injuries, typified and sanctioned in articles 147, 148, 149 and 150 [20].

Within the international legal framework, the United Nations (UN) created the Commission on the Juridical and Social Status of Women in 1946. On 18 December 1979, the Convention on the elimination of all forms of discrimination against women was approved and came into force in 1981. Nowadays, the UN seeks to eliminate gender-based violence, including FGM as a form of violence against women, and in turn, protect children’s rights [21,22,23].

According to Kaplan et al., there is currently no documentary evidence that FGM is performed in Spain, although the latest data show that there are 224,139 people from countries where FGM is practiced, with a female population of 57,259 people of which 16,869 are girls from 0 to 14 years of age, who may be at risk of suffering any type of FGM [24].

Health professionals, given the work they perform, represent an important factor when it comes to detecting possible cases of FGM as well as people at risk.

Therefore, with the aim of addressing this problem, we developed this study where we analyze the knowledge of health professionals regarding this argument as well as the factors that could be related to this issue such as profession, gender, training, etc.

## 2. Materials and Methods

A cross-sectional study was conducted with a self-administered online survey to Primary Healthcare and Specialized Healthcare with the following profiles: nursing, midwifery, general practice, pediatrics and gynecology–obstetrics.

Data were collected from October 2019 and January 2020 among professionals from the five provinces of Castilla-La Mancha (Albacete, Ciudad Real, Cuenca, Guadalajara, Toledo), a region where, according to data from the National Statistical Institute for the year 2017, 520 girls under the age of 15 resided in the territory with nationality of countries where FGM is practiced (INE, 2017).

The strategy of survey distribution consisted first, in elaborating an online version of the questionnaire elaborated by Kaplan et al. in 2009. Subsequently, from the information technology service of the Castilla la Mancha Health Service (SESCAM), the access link to the survey was distributed via email to professionals with the previously selected groups.

The survey collected information on sociodemographic variables (age, gender, profession, work place, territory of work and work experience in healthcare) degree of knowledge on FGM (identification and typology, reasons for this practice, countries in which it is carried out), degree of interest elicited (need or desire to know more on the subject, performance of educational activities or knowledge of protocols of guidelines of action), previous experience (care to patients from countries in which FGM is performed, detection of any case) and attitudes versus FGM (ignore, educate-sensitive, report to authorities) [23].

The surveys were anonymous so that participants could not be identified.

### Statistical Analysis

First, a descriptive analysis of the variables under study and the subsequent assessment of association between them was carried out. 

Secondly, to identify differences in knowledge, attitude and interest of the professionals, the total proportions of these variables were compared, also stratified by, age group, profession and gender with the chi-square test. 

To identify the characteristics of the professionals which may influence correct identification of the FGM, as well as the detection of cases and the attitude, a logistic regression model was performed, using age, profession, gender and training received as independent variables and the knowledge of typology and countries of prevalence of FGM, cases detected and attitudes towards FGM as dependent variables (performing four individual analysis for every dependent variable).

Statistical significance was considered at *p* < 0.05.

The analyses were performed using IBM SPSS Statistics for Windows, version 24 (IBM Corp., Armonk, N.Y., USA).

## 3. Results

In total, 1347 answers were received from a total of 12,906 surveys distributed. Just 1168 were included in the analysis: more than 70% of participants were women (71.7%, *n* = 838) and the mean age of women was slightly lower (45.5) than that of men (50.2). We excluded 179 answers because they were incomplete.

The professional groups that participated were mainly represented by nurses (58.8%, *n* 687), followed by family doctors (29.6%, *n =* 346).

Regarding the workplace, primary healthcare was the most representative proportion in the sample (73.3%, *n* = 857) and professional’s working in Toledo province represent the highest percentage of responders (28.7%, *n* = 335) closely followed by those from Ciudad Real (23.0%, *n* = 269).

More than 90% of participants indicated having a professional experience of more than 24 months (94.9%, *n* = 1109).

The sociodemographic and professional characteristics of participants are shown in Table 2.

Knowledge, attitudes and interest related to FMG are shown in Table 3 and are detailed by age, profession and gender in Supplementary File S1.

Just 14.0% (*n* = 163) of participants indicated that had received training in FGM: 50% of obstetricians (*n* = 1), 40% of midwives (*n* 19) followed by 20.5% of pediatricians (*n* = 16), 16.7% of gynecologists (*n* = 2), 13.3% of family doctors (*n* 46) and 11,5% of nurses (*n* = 79). 

In the group of professionals that correctly identified both the type and the country of FGM, 24 previously received training (11 nurses, 7 midwives and 6 pediatricians) while 41 did not (*p* < 0.001).

Professionals indicated having received training mostly from health services (*n* = 58, 35.6%) followed by those that indicated the option “others” (*n* = 52, 31.9%) and NGOs/associations (*n* = 51, 31.3%).

In total, 125 professionals (10.7%) correctly identified the existence of the three types of FGM, while the most frequent answer was the total removal of the clitoris (53%, *n* = 626). In this context, midwives were the group with the most accurate knowledge about the types of FGM (*p* < 0.001). No statistically significant differences were found between the different age groups nor differences based on gender.

If we focus on the identification of the countries where FGM is practiced, 20.5% (*n* = 240) of the total sample, correctly identified it. Again, midwives were those that better identified it (37.2%, *n* = 16, *p* < 0.05). No statistically significant differences were found between the different age groups nor between males and females.

A total of 396 participants (33.9%) indicated the correct answer about the legislation in Spain, while 317 (27.1%) indicated not knowing the existence of legislation. No statistically significant differences were found between the different age groups nor when comparing by gender. Midwives better identified it (65.1%, *n* = 28) followed by pediatricians (62.8%, *n* = 49), (*p* < 0.001).

A significant association was found between the reasons why FGM is performed and the age of the participants (*p* < 0.05). Concretely, the belief that FGM is due to tradition and costumes is more frequent among the group of professionals between 41 and 50 years old, while the religious reasons for the practice of FGM is more frequent among the youngest group. Analyzing by profession, the belief that FGM is performed due to hygienic and esthetic reasons and also in order to get better opportunities to get married is more frequent among midwives (*p* < 0.05).

A statistically significant difference was also found between the reasons why FGM is performed based on gender (*p* < 0.05): females more frequently affirmed that FGM is due to the belief of having better opportunities to get married when compared to males.

Just 5.4% (*n* = 63) of the sample answered that they had found a case of FGM during their professional practice. In proportion, separating by groups, midwives detected more cases (53.5%, *n* = 23). In the case of professionals that indicated that they had not detected any cases, the most frequent answer was that they had attended the population at risk but had not detected any case, 46% (*n* = 542), followed by those that indicated not to have attended the population at risk, 36.0% (*n* = 420), and lastly those indicating that in the framework of their activity it was not appropriate to ask about FGM 12.2% (*n* = 143).

The detection of cases was divided into detection in cases under 18 and cases over 18 years old. Eight cases (12.6%) were girls under 18 and 55 cases were over 18 (87.3%). Of these cases, 74.5% (*n* = 41) were detected during a physical examination, 14.5% (*n* = 8) during a clinical interview and the rest in other circumstances such as childbirth.

Regarding the detection of cases under 18, 50% (*n* = 4) were detected during a physical examination, 37.5% (*n* = 3) due to a third-party complaint and 12.5% (*n* = 1) due to other reasons.

After detecting a case, 31.7% (*n* = 20) of professionals decided to address the issue in the consulting room, 23.8% (*n* = 15) asked if they had any other daughters (to know if there were other girls at risk) and 27% (*n* = 17) asked other professionals. However, no one reported it to authorities and 31.7% (*n* = 20) of people ignored it.

Of the total sample, 8.6% (*n* = 101) indicated to know any protocol. Statistically significant differences were found between the different professional groups (*p* < 0.001) but not between the different age groups of the participants and genders. Midwives and pediatricians with 44.2% (*n* = 19) and 20.5% (*n* 16), respectively, were the most aware of these protocols. Additionally, obstetricians obtained a high percentage (50%), but due to the fact that just two people participated and one of them indicated to know it. For this reason, we cannot consider it as a significant result.

On the other side, the measures proposed by the professionals in order to prevent FGM presented statistically significant differences between the different age groups, professional groups as well as between gender groups. By age, the youngest group mainly selected the options “periodic checkup”, “exemplary judicial sentences” and “to train primary care professionals to carry out prevention” while professionals over 51 years old mostly selected the measure “prevent travel to risk countries”. However, when analyzing the answers by professional groups, gynecologists selected this last measure as well as that proposed “exemplary judicial sentences”. Finally, dividing by gender, the options “materials to address the issue with the families”, “intercultural mediation” and also the option “counselling the cases at risk”, were mainly selected by women (*p* < 0.05).

The characteristics of the professionals who may have influenced in the correct identification of FGM, the detection of cases and attitudes are shown in Supplementary File S2. 

When analyzing the different groups by age, no differences were found in knowledge or attitudes or gender.

By profession, midwives demonstrated better knowledge about the typology of FGM (OR = 4.3; CI 95% [2.1–8.8]) and greater probability of case detection (OR = 48.5 [21.3–110.1]).

Interestingly, professionals with specific training would identify better both the typology and the countries where FGM is practiced but did not present a greater probability to detect cases.

Finally, about the attitudes, gynecologists and midwives were those that mainly chose to discuss the issue in the outpatient clinic and to ask about other daughters that could be at risk (OR = 21.9; CI 95% [2.0–244.1]; OR = 14.9; CI 95% [1.4–160.7]) and (OR = 43.7; CI 95% [10.7–178.4]; OR = 28.9; CI 95% [7.6–110.7]), respectively, but midwives also selected the option of asking and coordinating to other professionals (OR = 83.2; CI 95% [19.6–352.5]).

## 4. Discussion

The results obtained from our study highlight the important problem surrounding FGM if we study the knowledge and skills of health professionals in the region of Castilla la Mancha.

There is a lack of knowledge about the types of FGM, countries where it is performed, legislation in Spain and the detection of cases. However, these are worrying data if we take into account that a significant percentage of professionals indicated attending population at risk because we could assume that many cases are not being diagnosed and many girls at risk may not be receiving the necessary support.

These results are in line with those reported by Kaplan et al. (2009). However, they indicated that their low rates of case detection were due to the fact that their study was conducted just in primary healthcare area, and our study also including the hospital environment did not obtain a higher level of detection [23]. 

A very low proportion of the participants indicated having received some type of training. However, this training showed a significant effect in the statistical analysis when identifying the types of FGM and countries at risk but the number of professionals identifying these data was higher among participants that indicated they did not receive any training. Therefore, some of the professionals participating in the study have overestimated their knowledge about FGM or the training they performed was not enough. This is in line with previous studies from countries reporting high risk of FGM [25,26,27,28].

The lack of training is also reported in the literature that shows that the strategies implemented in many countries against FGM are not adequate [29].

Therefore, this study shows the important need to improve the actions aimed to prevent and eradicate FGM in Castilla La Mancha.

In proportion, midwives were those that detected more cases, and most of them were identified during a physical exploration. Maternal and infant stages are optimal stages for the detection of cases because in some cases it is the main contact with the health system [30]. In the global analysis, it was found that midwives represented a bigger probability of finding cases maybe indicating that specific attention to women´s health facilitates closer contact with patients as well as a greater possibility of conducting interviews that include aspects oriented to the prevention and detection of FGM.

It is also important to bear in mind that when asking the professionals about the action they took, an important proportion ignored it and did not reported it to authorities. This could be due to the lack of knowledge about legislation in our country, because 27.1% indicated not knowing about it and just 33.9% indicated the correct answer. Therefore, this misinformation could be behind the fact of ignoring and not reporting the cases detected, also ignoring the obligation of professionals to report a criminal act that is punished in our country.

The proposals indicated by the professionals showed a bigger interest in developing activities with a closer contact with people, concretely, women selected mainly options such as counselling or intercultural mediation. They also considered the need of having more material to address the problem.

The WHO proposed, in a document published in 2001, including FGM in the curricula of several disciplines at university and high school level in order to improve this situation [31].

This could be a great solution, but also promoting a greater awareness of professionals in the health system, more multidisciplinary collaboration and more quality training could improve the situation described in the present study. For example, a similar situation was described in the work published by Kaplan et al. in 2009, where they indicated that after carrying out different educational activities in the healthcare centers, it was found an increase of FGM cases identified [23]. In a recent qualitative study developed over healthcare professionals in Castilla la Mancha, they concluded that the existence of a protocol of action and training could be the key tools to take into account to address this problem [32].

In the last decades, several studies have investigated the knowledge and abilities of healthcare professionals regarding FGM. For example, researchers from different European countries such as Jager and collaborators in Switzerland, Tammaddon and colleagues in Sweden or Leye and co-workers in Belgium among others, showed higher rates of case detection than those found in our study and also with respect to the results described by González-Timoneda et al. [33,34,35,36,37,38].

However, those studies show data collected at national level. In our country, there is still a lack of studies evaluating the real level of knowledge and attitudes of healthcare professionals regarding this argument. For this reason, further studies are certainly warranted to clarify all these aspects and to afront this violation of women’s human rights.

## 5. Conclusions

The present study demonstrates that there is a problem linked to the FGM in the healthcare professionals in Castilla la Mancha. Concretely, the professionals presented a lack of knowledge about important aspects related to that issue such as the typology and countries of prevalence, which means a limitation to detect cases at risk and to develop actions aimed at preventing and eradicating FGM.

It was also shown that the professional experience facilitates these abilities among the professionals.

There is also an over-evaluation of the participants responding that they have already received specific training because in the results, it was shown that the detection of cases was more frequent in people that indicated not having any training related to FGM and something similar happened when identifying basic aspects related to this issue such as the types of FGM and the countries where it is usually performed.

No effects were found when studying the differences in knowledge related to the professional experience. However, the midwives´ group was the most able to identify the information related to FGM. Furthermore, it was also the group that detected more cases.

Health professionals have a responsibility to have the knowledge and attitudes necessaries to detect cases of FGM as well as to know the necessary protocols to follow to refer these women and their families to professionals and specialized services that guarantee them adequate care based on a multidisciplinary, transcultural and positive approach that guarantees their safety and well-being.

Further studies and training programs are necessary to improve the healthcare professionals’ skills in the field of FGM.

## 6. Limitations of the Study

Regarding the limitations of the present study, some of the analyses developed did not show significance due to insufficient sample size in some of the professional groups such as midwives, obstetricians, and gynecologists. It could be more efficient to calculate the sample and randomize the distribution but calculate the representativeness of every professional group. However, due to the experimental design, and the impossibility of having the list of professionals before starting the study, the procedure was conducted in this way.

It should be also noted that some questions could lead to different interpretations and to different responses, but it is considered as a part of the variability included in the data.

## Figures and Tables

**Table 1:** WHO classification of FGM.

Type I: Partial or total removal of the clitoris and/or the prepuce (clitoridectomy) Type Ia: Removal of the clitoral hood or prepuce only Type Ib: Removal of the clitoris * with the prepuce
Type II: Partial or total removal of the clitoris and the labia minora, with or without excision of the labia majora (excision) Type IIa: Removal of the labia minora only Type IIb: Partial or total removal of the clitoris and the labia minora Type IIc: Partial or total removal of the clitoris, the labia minora and the labia majora
Type III: Narrowing of the vaginal orifice with creation of a covering seal by cutting and apposition the labia minora and/or the labia majora, with or without excision of the clitoris (infibulation) Type IIIa: Removal and apposition of the labia minora Type IIIb: Removal and apposition of the labia majora
Type IV: Unclassified. All other harmful procedures to the female genitalia for nonmedical purposes, for example, pricking, piercing, incising, scraping and cauterizing the genital area.

* When total removal of the clitoris is reported, it refers to the total removal of the glans of the clitoris

**Table 2:** Sociodemographic and professional characteristics of participants.

	*n*	%
Included Surveys	1168	-
Gender		
Women	838	71.7
Men	330	28.3
Age (years)		
20–40	335	28.7
41–50	384	32.9
>50	448	38.4
No answer	1	0.1
Professional group		
Nursing	687	58.8
Midwifery	43	3.7
Family medicine	346	29.6
Pediatrics	78	6.7
Gynecology	12	1.0
Obstetrics	2	0.2
Workplace		
Primary healthcare center	716	61.3
Local office	141	12.1
Hospital (emergency)	119	10.2
Hospital (delivery room)	24	2.1
Hospital (maternity)	19	1.6
Hospital (pediatrics)	43	3.7
Others	106	9.9
Territory of work		
Albacete	197	16.9
Ciudad Real	269	23.0
Cuenca	171	14.6
Guadalajara	196	16.8
Toledo	335	28.7
Work experience in healthcare		
3–11 months	29	2.5
12–23 months	30	2.6
>24 months	1109	94.9
Have received training	163	14.0
Training given by		
Security forces	1	0.1
Health services	58	5.0
NGO or associations	51	4.4
Governmental organizations	27	2.3
Others	52	4.5

**Table 3:** Knowledge, attitudes and interest related to FGM.

	*n*	%
Have received training	163	14.0
Correct identification		
Type of FGM	125	10.7
Countries	240	20.5
Legislation in Spain	396	33.9
Believe performed for		
Tradition	1049	89.8
Religion	621	53.2
Hygiene	18	1.5
Esthetic	16	1.4
Better opportunities to get married	120	10.3
Do not know	26	2.2
Proposals for prevention		
Periodic checkup	446	38.2
Prevent travel to risk countries	109	9.3
Sensitize parents	971	83.1
Exemplary judicial sentences	290	24.8
Report to authorities	350	30.0
Train primary care professionals to carry out prevention	915	78.3
Have detected some cases	63	5.4
Attitude		
Discuss in the outpatient clinic	20	1.7
Asked about other daughters	15	1.3
Asked other professionals	17	15
Report to authorities	0	0
Ignore	20	1.7
Know some protocol of action	101	8.6
Proposals to improve care and prevention		
Education	1067	91.4
Material for professionals	500	42.8
Material to discuss the issue with families	686	58.7
Intercultural mediation	607	52.0
More time to address it	630	53.9
Counseling	708	60.6
Greater police intervention	173	14.8

## Data Availability

The datasets used and analyzed during the current study are available from the corresponding author on reasonable request.

## Supplementary Materials

The following are available online at https://www.mdpi.com/article/10.3390/healthcare9080974/s1, Supplementary File S1, Supplementary File S2.
