# Supplementary material for: Female Genital Mutilation: Knowledge and Skills of Health Professionals
Source: Healthcare (Basel). 2021 Jul 31;9(8):974. doi: 10.3390/healthcare9080974 (PMC8392609; doi:10.3390/healthcare9080974)
Supplement: Supplementary file 1 [file healthcare-09-00974-s001.zip › Additional file 1.pdf]

	Age						
	20-40 (N335)		41-50 (384)		>50 (448)		p
	N	%	N	%	N	%	
**Have received training**	43	12.8	59	15.4	61	13.6	
**Correct identification**							
Type of FGM	30	8.9	47	12.2	48	10.7	
Countries	77	23.0	78	20.3	85	19.0	
Legislation in Spain	101	30.1	126	32.8	169	37.7	
**Believe performed for**							
Tradition	289	86.3	356	92.7	403	90.0	**0.017**
Religion	200	59.7	209	54.4	212	47.3	**0.002**
Hygiene	6	1.8	4	1.0	8	1.8	
Esthetic	6	1.8	6	1.6	4	0.9	
Better opportunities to get married	33	9.9	48	12.5	39	8.7	

Don´t know	9	2.7	7	1.8	10	2.2	
**Proposals for prevention**							
Periodic checkup	150	44.8	147	38.3	149	33.3	**0.005**
Prevent travel to risk countries	20	6.0	33	8.6	56	12.5	**0.007**
Sensitize parents	275	82.0	326	84.9	369	82.4	
Exemplary judicial sentences	104	31.0	92	24.0	94	21.0	**0.005**
Report to authorities	275	82.1	306	79.7	333	74.3	
Train primary care professionals to carry out prevention	107	31.9	116	30.2	127	28.3	**0.024**
**Have detected some case**	23	6.9	18	4.7	22	4.9	
**Attitude**							
Discuss in the outpatient clinic	7	2.1	6	1.6	7	1.6	
Asked about other daughters	3	0.9	6	1.6	6	1.3	

Asked other professionals	7	2.1	7	1.8	3	0.7	
Report to authorities	0	0	0	0	0	0	
Ignore	8	2.4	5	1.3	7	1.6	
**Know some protocol of action**	26	7.8	38	9.9	36	8.0	
**Proposals to improve care and prevention**							
Education	316	94.3	351	91.4	399	89.1	**0.035**
Material for professionals	173	51.6	170	44.3	157	35.0	**<0.001**
Material to discuss the issue with families	224	66.9	238	62.0	224	50.0	**<0.001**
Intercultural mediation	197	58.8	205	53.4	205	45.7	**0.001**
More time to address it	185	55.2	226	58.8	218	48.7	**0.011**
Counseling	228	68.1	244	63.5	235	52.4	**<0.001**
Greater police intervention	60	17.9	55	14.3	58	12.9	

	Profession												
	N (N 687)		G (N 12)		Fm (N 346)		Mw (N 43)		O (N 2)		P (N 78)		p
	N	%	N	%	N	%	N	%	N	%	N	%	
**Have received training**	79	11.5	2	16.7	46	13.3	19	44.2	1	50	16	20.5	**<0.001**
**Correct identification**													
Type of FGM	61	8.9	5	41.7	32	9.3	16	37.2	0	0	11	14.1	**<0.001**
Countries	138	20.1	1	8.3	59	17.1	16	37.2	0	0	26	33.3	**0.002**
Legislation in Spain	191	27.8	8	66.7	119	34.4	28	65.1	1	50	49	62.8	**<0.001**
**Believe performed for**													
Tradition	611	88.9	12	100	346	100	43	100	2	100	78	100	
Religion	360	52.4	7	58.3	182	52.6	24	55.8	1	50	47	60.3	
Hygiene	4	0.6	0	0	4	1.16	4	9.3	0	0	6	7.7	**<0.001**

Esthetic	5	0.7	0	0	2	0.6	4	9.3	0	0	5	6.4	**<0.001**
Better opportunities to get married	47	6.8	3	3	40	11.6	14	32.5	0	0	16	20.5	**<0.001**
Don´t know	19	2.8	0	0	6	1.73	0	0	0	0	1	1.28	
**Proposals for prevention**													
Periodic checkup	252	36.7	5	41.7	128	37.0	19	44.2	1	50	41	52.6	
Prevent travel to risk countries	68	9.9	4	33.3	31	9.0	3	7.0	1	50	2	2.5	**0.005**
Sensitize parents	577	84.0	10	83.3	275	79.5	38	88.4	2	100	69	88.5	
Exemplary judicial sentences	187	27.2	5	41.7	69	19.9	14	32.5	0	0	15	19.2	**0.039**
Train primary care professionals to carry out prevention	535	77.9	8	66.7	264	76.3	41	95.3	2	100	65	83.3	
Report to authorities	197	28.7	5	41.7	110	31.8	18	41.8	0	0	20	25.6	

**Have detected some case**	16	2.3	3	25.0	15	4.3	23	53.5	1	50.0	5	6.4	**<0.001**
**Attitude**													
Discuss in the outpatient clinic	3	0.4	1	8.3	5	1.4	9	21.0	1	50	1	1.3	**<0.001**
Asked about other daughters	4	0.6	1	8.3	1	0.3	7	16.3	1	50	1	1.3	**<0.001**
Asked other professionals	3	0.4	0	0	1	0.3	11	25.6	1	50	1	1.3	**<0.001**
Report to authorities	0	0	0	0	0	0	0	0	0	0	0	0	
Ignore	5	0.7	2	16.7	6	1.7	7	16.3	0	0	0	0	**<0.001**
**Know some protocol of action**	42	6.1	1	8.3	25	7.2	8	18.6	1	50	24	30.8	**<0.001**
**Proposals to improve care and prevention**													
Education	639	93.0	10	83.3	309	89.3	40	93.0	2	100	67	85.9	
Material for professionals	282	41.0	5	41.7	148	42.8	24	55.8	0	0	41	52.6	

Material to discuss the issue with families	409	59.5	7	58.5	188	54.3	33	76.7	0	0	49	62.8	**0.034**
Intercultural mediation	365	53.1	7	58.5	157	45.3	34	79.1	0	0	44	56.4	**0.001**
More time to address it	351	60.8	7	58.5	189	54.6	24	55.8	1	50	58	74.3	**0.008**
Counseling	418	14.8	6	50	200	57.8	33	76.4	2	100	49	62.8	
Greater police intervention	102	14.8	3	25	47	13.6	12	27.9	0	0	9	11.5	

	**Female**	**%**	**Male**	**%**	**p**
**Have received training**	118	14,1	45	13,6	
**Correct identification**	43	5,1	9	2,7	
Type of FGM	97	11,6	28	8,5	
Countries	179	21,4	61	18,5	
Legislation in Spain	286	34,1	110	33,3	
**Believe performed for**					
Tradition	751	89,6	298	90,3	
Religion	446	53,2	175	53,0	
Hygiene	12	1,4	6	1,8	
Esthetic	9	1,1	7	2,1	
Better opportunities to get married	97	11,6	23	7,0	**0,020**

Don´t know	18	2,2	8	2,4	
**Proposals for prevention**					
Periodic checkup	330	39,4	116	35,2	
Prevent travel to risk countries	71	8,5	38	11,5	
Sensitize parents	710	84,7	261	79,1	**0,021**
Exemplary judicial sentences	211	25,2	79	23,9	
Report to authorities	242	28,9	108	32,7	
Train primary care professionals to carry out prevention	679	81,0	236	71,5	**<0,01**
**Have detected some case**	48	5,7	15	4,5	
**Attitude**					
Discuss in the outpatient clinic	16	1,9	4	1,21	
Asked about other daughters	12	1,4	3	0,90	
Asked other professionals	12	1,4	5	1,5	
Report to authorities	0	0	0	0	
Ignore	17	2,0	3	0,9	
**Know some protocol of action**	71	8,5	30	9,0	
**Proposals to improve care and prevention**					
Education	773	92,2	294	89,0	
Material for professionals	370	44,2	130	39,3	
Material to discuss the issue with families	525	62,6	161	48,8	**<0,01**
Intercultural mediation	453	54,0	154	46,7	**0.023**
More time to address it	457	54.5	173	52.4	
Counseling	529	63.1	179	54.2	**0.005**
Greater police intervention	124	14.8	49	5.84	
