# Supplementary material for: Female Genital Mutilation: Knowledge and Skills of Health Professionals
Source: Healthcare (Basel). 2021 Jul 31;9(8):974. doi: 10.3390/healthcare9080974 (PMC8392609; doi:10.3390/healthcare9080974)
Supplement: Supplementary file 1 [file healthcare-09-00974-s001.zip › Additional file 2.pdf]

Characteristic	knowledge			Attitudes				
	Typology OR (CI)	Countries of prevalence OR (CI)	Cases detected OR (CI)	Discuss in the outpatient clinic OR (CI)	Asked about other daughters OR (CI)	Asked other professionals OR (CI)	Report to authorities OR (CI)	Ignore OR (CI)
**Age**								
20-40	1	1	1	1	1	1	1	1
41-50	1.5 (0.9-2.5)	0.8 (0.6-1.2)	0.6 (0.3-1.3)	0.7 (0.2-2.4)	2.1 (0.5-9.2)	0.9 (0.3-2.9)	0	0.5 (0.2-1.8)
>50	1.4 (0.8-2.3)	0.8 (0.5-1.1)	0.8 (0.4-1.5)	1.0 (0.3-3.3)	2.5 (0.5-11.6)	0.3 (0.1-1.7)	0	0.8 (0.3-2.4)
**Profession**								
Nursing	1	1	1	1	1	1	1	1
Gynaecologist	8.5 (2.5-29.1)*	0.36 (0.04-2.8)	14.7 (3.5-61.6)	21.9 (2.0-244.1)*	14.9 (1.4-160.7)*	0	0	39.5 (6.2-250.9)
Family medicine	1.0 (0.7-1.7)	0.84 (0.6-1.2)	2.0 (1.0-4.3)*	3.7 (0.8- 16.0)	0.5 (0.5-4.3)	0.6 (0.1-5.8)	0	3.0 (0.9-113.7)
Midwive	4.3(2.1-8.8)*	1.7 (0.9-3.4)	48.5 (21.3-110.1)*	43.7 (10.7-178.4)*	28.9 (7.6-110.7)*	83.2 (19.6-352.5)*	0	32.0 (9.0-113.2)
Obstetrician	0	0	0	0	0	0	0	0
Paediatrician	1.5(0.7-3.0)	1.9 (1.4-3.2)*	3.0 (1.0-8.5)	2.7 (0.3-26.8)	1.9 (0.2-17.4)	3.2 (0.3-31.9)	0	0
**Gender**								
Male	1	1	1	1	1	1	1	1
Female	0.7(0.4-1.1)	0.9 (0.6-1.3)	0.8 (0.4-1.7)	0.6 (0.2-2.2)	0.8 (0.2-3.3)	3.0 (0.8-11.4)	0	0.4 (0.1-1.5)
**Training**								
Training not received	1	1	1	1	1	1	1	1
Training received	3.0 (1.9-4.7)*	2.3 (1.6-3.4)*	1.0 (0.5-2.1)	2.5 (0.9-7.0)	1.9 (0.6-6.3)	1.2 (0.4-4.2)	0	0.5 (0.1-1.9)

Results are expressed using Odds Ratio and CI 95%

* $p < 0.05$ on comparison with the reference category
